# Exploring Zirconia Adhesion: Pre and Postsintering Physical Surface Treatment, Chemical Treatment, and Cement Interactions

**DOI:** 10.1155/2024/5394652

**Published:** 2024-08-22

**Authors:** Flávia Gonçalves, Mirko Dennys Ayala-Perez, Francisco Carlos dos Santos Reis, Walter Gomes Miranda-Júnior, Letícia Cristina Cidreira Boaro

**Affiliations:** ^1^ Faculdade de Odontologia Universidade Santo Amaro, Av. Prof. Eneas de Siqueira Neto, 340 04829-900, São Paulo, SP, Brazil; ^2^ Departamento de Biomateriais e Biologia Oral Faculdade de Odontologia da Universidade de São Paulo, Av. Prof. Lineu Prestes, 2222 05508-000, São Paulo, SP, Brazil; ^3^ College of Dentistry University of Saskatchewan, 105 Wiggins Rd, Saskatoon, SK S7N 5E5, Canada

**Keywords:** adhesives, resin cements, sandblasting, shear strength, zirconia

## Abstract

**Background:** Adhesion to zirconia remains a significant dental challenge. This study is aimed at assessing the bond strength of zirconia based on surface treatment with pre or postsintering sandblasting associated with different chemical treatments and resin cements.

**Methods:** Zirconia blocks were divided into 12 experimental groups based on the surface treatment (presintering sandblasting or postsintering sandblasting/tribochemical abrasion treatment), chemical treatment (none, Single Bond Universal, or Signum Zirconia Bond), and choice of cement (Panavia F or RelyX™ U200). The bond strength was measured by shear tests using a universal testing machine. The fracture analysis was performed using stereomicroscopy. Data were analyzed using three-way ANOVA and Tukey's test (*α* = 5%).

**Results:** Triple and double factor's interactions were not significant (*p* > 0.05). Regarding the surface treatment factor, the bond strength following postsintering sandblasting treatment associated with tribochemical abrasion (9.15 ± 3.62 MPa) was significantly higher than presintering sandblasting treatment (5.24 ± 3.53 MPa). Concerning the chemical treatment factor, bond strengths were ranked as follows: Signum Zirconia Bond > Single Bond Universal > no treatment. The bond strength of the resin cements did not differ among them. Most fractures (67%) were classified as adhesive, and 32% were categorized as mixed fractures.

**Conclusion:** Surface treatment via postsintering sandblasting combined with tribochemical abrasion demonstrated superior efficacy than in presintering sandblasting. Additionally, chemical treatment with zirconia primer increased the bond strength of zirconia irrespective of the surface physical treatment.

## 1. Introduction

Currently, the use of ceramic materials in dentistry has continued to grow owing to their high aesthetic properties, mechanical properties, biocompatibility, and color stability [1, 2]. Zirconia, a ceramic with the highest mechanical properties, is widely used in dentistry for monolithic restorations, frameworks for fixed prostheses, intraradicular posts, and prosthetic components for dental implants [3, 4].

Unlike traditional feldspathic ceramics and lithium disilicate ceramics, zirconia does not contain a glass phase in its microstructure. As a result, etching with hydrofluoric acid does not alter the roughness of the material or increase the bond strength values [5–7]. Furthermore, the use of silane does not enhance the bond strength with zirconia [8], because silica is required for the chemical silanization reaction [7].

However, various strategies have been employed to improve the bond strength of zirconia, including both chemical and mechanical retention [9, 10]. Although there is no standardized effective protocol for zirconia adhesive luting, the use of primers containing 10-methacryloyloxydecyl dihydrogen phosphate (MDP) monomer molecules and techniques, such as sandblasting and tribochemical treatment, has shown promising results with high bond strength [10, 11].

MDP is a functional monomer that is used in adhesives and ceramic primers. The phosphate and phosphonate groups present in the MDP molecules react with zirconia dioxide, forming primary bonds (P-O-Zr) that enhance the bond strength of the material. However, studies have indicated that the presence of other components added to the primer or adhesive in combination with MDP can compromise bond strength [12].

Sandblasting induces superficial roughness in zirconia, thereby promoting its mechanical retention. The effectiveness of sandblasting depends on factors such as the type of abrasive, pressure, and particle size, as well as the phase transformation in Y-TZP zirconia from a tetragonal to a monoclinic molecular structure [13], which improves the toughness of the material but can also cause damage to the zirconia structure [14].

On the other hand, tribochemical abrasion treatment involves sandblasting the surface of restorations using alumina coated with silica particles (Rocatec® 3 M ESPE). Because of the pressure applied during sandblasting, silica became embedded on the ceramic surface. This process creates micromechanical retention and facilitates chemical interactions between the silica and silane, as well as specific adhesives for zirconia, thereby improving the bond strength [9]. However, concerns have been raised regarding the long-term stability of the bond between silica and zirconia [12, 15] and the impact of sandblasting on the zirconia fracture strength.

Therefore, despite some success in the adhesion of zirconia, such treatments still present limitations, and new strategies are needed. One study has observed that the use of presintering sandblasting has improved the shear strength between porcelain and zirconia [16] and other showed similar shear strength [17]; this difference is apparently due to the system test used and the sandblasting pressure used; however, no study has evaluated the effect of the presintering treatment on the bond strength of zirconia to resin cement.

Therefore, the aim of this study was to evaluate the bond strength of zirconia in relation to sandblasting treatment before or after sintering, to the chemical treatment of the surface, and the type of resin cement used, through an *in vitro* investigation. The null hypothesis posits that shear strength is not influenced by (1) sandblasting and/or tribochemical abrasion treatments (2), chemical surface treatment, or (3) composition of the resin cement.

## 2. Material and Methods

### 2.1. Specimen Preparation

Zirconia blocks (Lava, 3 M-ESPE St. Paul, MN, USA) were sectioned with a diamond disc mounted on a high-precision cutter (Isomet 1000, Buehler, Germany) to obtain specimens of 7 × 7 × 3 mm^3^ (*n* = 120). All specimens were polished with abrasive aluminum oxide sandpaper with 1200 grit (Norton Brasil) over continuous water spraying. The samples were randomly divided into 12 groups (Table 1), according to the sandblasting (presintering sandblasting or postsintering sandblasting/ tribochemical abrasion), the primer used (none, Signum Zirconia Bond, or Single Bond Universal), and resin cement (RelyX™ U 200 or Panavia F). The compositions of the primers and resin cement used are listed in Table 2.

In the presintering sandblasting group (*n* = 60), the specimens were blasted with 150 *μ*m aluminum oxide according to the following parameters: distance of 10 mm, 45° of angulation, the pressure of 2.8 bar for 10 s, and sintered in a zirconia oven (Zyrcomat, Vita, Zanhfabrik, Alemanha) with a sintering cycle of 1530°C for 2 h, with an average heating rate of 25°C per minute and a cooling time of 7.5 h. The postsintering sandblasting groups (*n* = 60) were blasted after sintering using the same pattern of sintering and sandblasting with aluminum oxide and then subjected to tribochemical abrasion treatment with 30 to 110 *μ*m silica-coated aluminum oxide (Al_2_O_3_) particles (Rocatec®, 3 M ESPE). The specimens of all groups were included in PVC cylinders (25 × 10 mm) using chemically activated acrylic resin (Jet–Artigos Odontológicos Clássico Ltda., Brazil), isolating the treated face with a water-soluble gel, and leaving it free for the adhesion procedure.

The chemical agents (Signum Zirconia Bond or Single Bond Universal) were applied according to the manufacturer's instructions. Briefly, a homogeneous layer of Signum Zirconia Bond I was applied on the surface and dried slightly with air, Signum Zirconia Bond II was applied in thin layers and cured for 40 s using a dimmable halogen light curing light model VIP Junior™ (Bisco. Inc.,1100 W. Irving Park Rd., Schaumburg, USA) with an irradiance of 450 mW/cm^2^. Single Bond Universal was applied in one layer, the solvent was evaporated for 20 s, slightly with air, and the adhesive was polymerized for 40 s using the same curing device.

**Figure 1:**
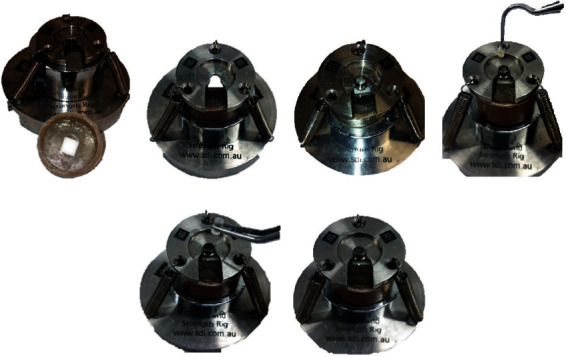
Specimen configuration. (a) Standardization device and specimen included in PVC tube with acrylic resin; (b) specimen inserted in the support device; (c) metal cylinder positioned, ready for cementation; (d) insertion of resin cement; (e) plastic cylinder positioned to remove excess resin cement and standardize the final amount of material; (f) removal of excess cement and correct position of the plastic cylinder.

After the chemical agent application, the specimens were positioned in a standardization device (Southern Dental Industries, Victoria, Australia) [18], which consisted of the following: two dense metallic plates joined by three springs to hold and center the PVC cylinders that contained the specimen; a small hollow metallic cylinder, with an internal diameter of 3.5 mm, where a transparent acrylic pin with 3 mm diameter, 3.8 mm in height, was introduced in order left a 0.2 mm span for the cement filling (Figures 1(a), 1(b), and 1(c)).

The resin cement was mixed and placed inside the hollow cylinder, according to the manufacturer's instructions (Figure 1(d). The acrylic pin was also placed inside the cylinder to remove excess cement (Figures 1(e) and 1(f)). The set was light-cured for 20 s for both resin cements. In the groups with Panavia F cement, Oxiguard II was applied for 3 min to protect the specimens and then washed with running water for 10 s to remove the product, as recommended by the manufacturer. All specimens were stored in a humid atmosphere, without direct contact with water, in the oven at 37°C for 24 h.

### 2.2. Shear Test and Fracture Analysis

**Figure 2:**
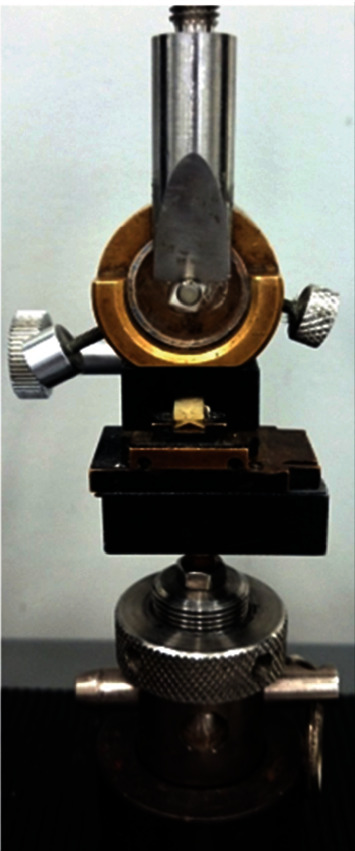
Shear test setup.

The shear strength tests were performed using a universal testing machine (KRATOS, LKC3 – USB, Brazil). The PVC cylinder containing the sample was fixed in a metallic matrix, and the straight-edge knife was kept parallel to the adhesive interface (Figure 2). The shear force was applied until a fracture occurred between cement and zirconia, at a constant speed of 0.5 mm/min [18].

After fracture, all specimens were evaluated to determine the fracture type, using a stereomicroscope at 40x magnification (model SZ61, Olympus Inc., Tokyo Japan) equipped with a CCD camera (Q-Color 3, Olympus). The fractures were classified as follows: adhesive failure between cement/zirconia when the zirconia surface was visible, cohesive failure in cement when the fractured surface was covered with cement, and mixed failure when adhesive and cohesive failures were observed on the same fracture surface.

### 2.3. Statistical Analyses

Data were submitted to normality (Shapiro–Wilk) and homoscedastic tests (Levene's test). Three-way ANOVA and Tukey's test were performed (factors: sandblasting, chemical agents, and cements), with a global level of significance of 95% (*α* = 0.05).

## 3. Results

The shear strength data for the experimental groups are listed in Table 3. Statistical analysis showed no significance in the triple interaction (*p* = 0.278) or in any of the double interactions (sandblasting × cement, *p* = 0.538; chemical treatment × cement, *p* = 0.141; sandblasting × chemical treatment, *p* = 0.965). When the factors were analyzed individually, the cement factor was also not significant (*p* = 0.227). For the sandblasting factor, the shear strength of sandblsting/tribochemical abrasion postsintering was statistically greater than the shear strength of sandblasting presintering (Table 3). Regarding the chemical treatment factor, the shear strength was statistically different for the three conditions (*p* < 0.001), Signum Zirconia Bond presented the highest shear strength, followed by Single Bond Universal, and the control with no chemical treatment showed the lowest shear strength (Table 3).

The analysis of fracture mode showed that adhesive fractures represented the majority of samples (67%), whereas only 1% presented cohesive fractures (Table 4).

## 4. Discussion

The null hypotheses were partially rejected, although no statistical difference was observed regarding resin cement, the chemical treatment and physical surface treatment significantly affected the shear strength.

Sandblasting with abrasive particles, mainly with Al_2_O_3_, increases the surface roughness of zirconia and it is easy to use in the dental office and simple to apply [9, 19, 20], the particles used for abrasion must range in size from 50 to 250 *μ*m [19]. Although studies have reported improvements in shear strength from zirconia to porcelain using the presintering sandblasting [16] or the absence of statistical differences in the sandblasting postsintering group [17], in the present study, the specimens that received presintered aluminum oxide sandblasting (150 *μ*m) obtained lower bond strength results than the group that received postsintering sandblasting, which is in agreement with studies that evaluated the effect of sandblasting pre and postsintering on biaxial flexural strength and four-point flexural strength [13, 21]. Although sandblasting presintering increases the surface roughness [16, 17], it does not necessarily improve the bond strength [16, 17]. A study that evaluated zirconia and porcelain adhesion observed that the pressure used in sandblasting is an important factor to guide the shear strength of the materials, an increase of 0.2 to 0.5 MPa in sandblasting pressure increases the surface roughness and improves the bond strength in materials with sandblasting postsintering from 25 to 30 MPa, making them statistical lower or similar to materials with sandblasting presintering materials (0.2 MPa pressure) [16]. In this study, the sandblasting pressure used was 2.8 bar (0.28 MPa), and a lower bond strength was observed between zirconia and resin cement in presandblasting samples. However, it should be noted that the postsintering treatment applied was sandblasting associated with tribochemical abrasion; thus, the last one can explain the increase in bond strength in these posttreated samples. The addition of a thin layer of silica on the treated surface roughens the zirconia surface and makes it chemically active, making it more receptive to chemical bonding through silane coupling agents [22, 23] and promoting chemical reactions with adhesives containing MDP [24]. In vitro studies have shown that the combination of mechanical pretreatment with tribochemical silica sandblasting (CoJet, 3 M ESPE; Seefeld, Germany) and chemical pretreatment with ceramic primers (Clearfil Ceramic Primer, Kuraray Noritake; Tokyo, Japan) provided the highest bonding effectiveness to dental zirconia [22].

The study design did not include a group with tribochemical silica abrasion associated with sandblasting conducted presintering, since the silica degrades, during the sintering process [25, 26]. Studies have reported that silica (SiO_2_) degrades, generating defects and weight loss at high temperatures, by the reduction of SiO_2_ in Si in a vacuum atmosphere (from 950°C to 1250°C) [25], or by the formation of Si(OH)_4_ gaseous, in the presence of humidity and high pressure (from 1200°C to 1350°C) [26]. Because the zirconia sintering temperature was 1530°C, the silica incorporated using tribochemical abrasion before sintering would be degraded, and the surface treatment would be ineffective. Therefore, only sandblasting presintering was compared as an alternative treatment, and tribochemical postsintering was maintained because it improved the adhesive strength in sandblasted samples [22].

Regarding the chemical treatments used, the Signum Zirconia Bond presented higher bond strength than Single Bone Universal, and no treatment showed lower bond strength than both chemical treatments used. Both materials used presented higher shear strength than the no-treated group, demonstrating the importance of associating a chemical treatment with the sandblasted surface [27], both before and after sintering. Signum Zirconia Bond is a specific primer for zirconia, whereas the Single bond Universal is a universal adhesive. Although both materials present MDP in this composition, which is an important molecule to establish the zirconia adhesion through the primary bond to zirconia dioxide [12], some studies highlighted the presence of other compounds in the adhesive bottle that can reduce the bond strength to zirconia [12, 28] in two ways: first, the silane present in the universal adhesive can compete with zirconia oxide to bond with phosphate groups [29]; and second, the water present in the adhesive can decrease the affinity of MDP to the zirconia, since it is insoluble in water [12, 28], and a better performance of MDP has been described in adhesives with acetone [28].

The resin cement used did not affect the bond strength in the present study, and in fact, a systematic review that evaluated adhesion to zirconia concluded that the choice of resin cement is less relevant than the surface treatment [10]. In addition, both resin cements used present 10-MDP molecules in their formulations that can explain the similar bond strength of both. Although some studies have shown that resin cements can affect significantly the bond strength to zirconia [30, 31], this can be associated with different performances depending on the ceramic material and surface treatment used [32, 33].

Regarding the fracture mode, when aluminum oxide sandblasting was associated with Panavia F cement, cohesive failure was 95% [34], another study presented a 100% adhesive failure when resin cements were used regardless of the surface treatment [35], and adhesive containing MDP presented mixed failure from 60% to 95% [9]. The present study presented a majority of adhesive failures (66.8%), mixed failures around 32.5%, and a very low frequency of cohesive failure (0.83%). Owing to the wide variability of data from the literature regarding the failure mode, it was possible to conclude that other factors besides the material composition can affect the data, such as methodology parameters, settings related to polymerization time and device, and handling of the materials, among others.

## 5. Conclusion

Within the limitations of this study, it can be concluded that presintering sandblasting was not effective in improving the bond strength to zirconia when compared to sandblasting postsintering associated with tribochemical abrasion, independently of chemical treatment or resin cement used. In addition, the use of zirconia primers improved the bond strength in relation to universal adhesives independently of the type of sandblasting or resin cement; therefore, clinicians should pay attention to this point. Because sandblasting can be affected by the size of alumina particles and by pressure, these factors should be analyzed in future studies, as well as their effects on roughness and flexural strength of the material in the short and long term, to check whether sandblasting presintering can be an alternative treatment to improve the adhesion in zirconia.

## Figures and Tables

**Table 1:** Experimental groups evaluated.

	**Sandblasting**	**Chemical treatment**	**Cement**
1	Presintering sandblasting	Signum Zirconia Bond	RelyX™ U 200
2			Panavia F
3		Single Bond Universal	RelyX™ U 200
4			Panavia F
5		None	RelyX™ U 200
6			Panavia F

7	Postsintering sandblasting/Tribochemical abrasion	Signum Zirconia Bond	RelyX™ U 200
8			Panavia F
9		Single Bond Universal	RelyX™ U 200
10			Panavia F
11		None	RelyX™ U 200
12			Panavia F

**Table 2:** Material composition according to the manufacturing.

**Material**	**Composition**
Signum Zirconia Bond I	Acetone (>90%), 10-(phosphonooxy)decyl methacrylate (0 a 0.5%), and ácido acético (0 a 0.5%)
Signum Zirconia Bond II	Metacrilato de metila (50 a 75%), 7,7,9 (ou 7,9,9)-trimetil-4,13-dioxo-3,14-dioxa-5,12-diazahexadecano-1,16-diil bismetacrilato (25 a 50%), óxido de difenil(2,4,6-trimetilbenzoil)fosfina (2.5 a 3%), and tert-butyl perbenzoate (0.1 a 0.25%)
Single Bond Universal	MDP phosphate monomer, dimethacrylate resins, vitrebond™ copolymer, filler, ethanol, water, initiators, and silane
RelyX™ U 200	Base paste: methacrylate monomers containing phosphoric acid groups, methacrylate monomers, silanated fillers, initiator components, stabilizer, and rheological additives Catalyst paste: methacrylate monomers, alkaline (basic) fillers, silanated fillers, initiator components, stabilizer, pigments, and rheological additives
Panavia F	A paste: 10-methacryloyloxidecyl dihydrogen phosphate (MDP), hydrophobic aromatic dimethacrylate, hydrophobic aliphatic dimethacrylate, hydrophilic aliphatic dimethacrylate, silanized silica particle, silanized colloidal silica, dl-camphorquinone, catalysts, and initiators B paste: hydrophobic aromatic dimethacrylate, hydrophobic aliphatic dimethacrylate, hydrophilic aliphatic dimethacrylate, silanized barium glass particle, surface treated sodium fluoride, catalysts, accelerators, and pigments. OXYGUARD II: glycerol, polyethylene glycol, accelerating catalysts, and dye.

**Table 3:** Mean and standard deviation of bond strength (MPa) of samples with sandblasting presintering or sandblasting associated with tribochemical abrasion postsintering tested with two resin cements and two or no chemical treatments.

**Chemical agent**					**Mean/chemical treatment factor** ^∗^
	**Sandblasting presintering**		**Sandblasting postsintering/tribochemical abrasion**		
	**RelyX™ U 200**	**Panavia F**	**RelyX™ U 200**	**Panavia F**	
Signum Zirconia Bond	8.3 (1.8)	8.6 (3.1)	13.1 (2.6	11.6 (4.0)	10.42 (3.54)^a^
Single Bond Universal	5.5 (2.1)	5.9 (2.2)	8.6 (1.9)	10.3 (2.1)	7.55 (2.83)^b^
None	0.8 (0.9)	2.4 (2.1)	5.3 (0.6)	6.0 (1.1)	3.63 (2.47)^c^
Mean/sandblasting factor^∗^	5.24 (3.53)^b^		9.15 (3.62)^a^		—

^*^At the same mean column or mean line, values followed by different lowercase letters represent statistical differences (*p* < 0.05). There were no statistically significant differences (*p* > 0.05) for the two cements used (independent of the conditions tested).

**Table 4:** Frequency (%) of the types of fracture.

**Fracture**	**Frequency (%)**
Adhesive	67
Cohesive	1
Mixed	32

## Data Availability

The data that support the findings of this study are available from the corresponding author upon reasonable request.
